# All-optical dual photoacoustic and optical coherence tomography intravascular probe

**DOI:** 10.1016/j.pacs.2018.07.002

**Published:** 2018-07-24

**Authors:** Sunish J. Mathews, Callum Little, Christopher D. Loder, Roby D. Rakhit, Wenfeng Xia, Edward Z. Zhang, Paul C. Beard, Malcolm C. Finlay, Adrien E. Desjardins

**Affiliations:** a Department of Medical Physics and Biomedical Engineering, University College London, UK; b Wellcome/EPSRC Centre for Interventional and Surgical Sciences, University College London, UK; c Department of Cardiology, Royal Free Hospital, London, UK; d William Harvey Cardiovascular Research Institute, Queen Mary University of London, UK; e Barts Heart Centre, London, UK

**Keywords:** Endoscopic imaging, Intravascular photoacoustic imaging, Optical coherence tomography, All-optical intravascular catheter, Fiber optic ultrasound sensor

## Abstract

Intravascular imaging in percutaneous coronary interventions can be an invaluable tool in the treatment of coronary artery disease. It is of significant interest to provide molecular imaging contrast that is complementary to structural contrast provided by optical coherence tomography (OCT) and intravascular ultrasound imaging (IVUS). In this study, we developed a dual-modality intravascular imaging probe comprising a commercial OCT catheter and a high sensitivity fiber optic ultrasound sensor, to provide both photoacoustic (PA) and OCT imaging. With PA imaging, the lateral resolution varied from 18 μm to 40 μm; the axial resolution was consistently in the vicinity of 45 μm. We demonstrated the clinical potential of the probe with 2-D circumferential PA and OCT imaging, and with multispectral PA imaging.

Intravascular (IV) imaging is widely used to guide treatment of coronary artery disease (CAD) [1]. Optical coherence tomography (OCT), also known as optical frequency domain imaging (OFDI), can provide valuable information about plaque composition and features which convey risk of plaque rupture, thereby guiding the deployment of intracoronary stenting. OCT has spatial resolution that is sufficiently high to visualise individual cells in plaque, such as macrophages [2], but it can often be challenging to measure lipid plaque burden due to the limited imaging depth in tissue (typically 1–1.5 mm). Moreover, OCT does not provide positive molecular contrast for lipid, so that lipid-rich plaque can frequently be devoid of contrast. In contrast, optical spectroscopy can provide detailed information about plaque composition. Infrared spectroscopy, Raman spectroscopy, and near infrared fluorescence molecular imaging have been shown to provide clinically-relevant information, but in general they do not allow for signals to be resolved in depth [3]. This limitation of some spectroscopic methods can be prominent when assessing lipid plaque burden. Photoacoustic (PA) imaging, in which ultrasound (US) waves are generated in tissue using pulsed excitation light, can provide depth-resolved intravascular imaging with molecular contrast for lipids, at depths of up to 4 mm [4]. As such, PA imaging has strong potential as an imaging modality complementary to OCT. Dual modality approaches combining PA and OCT have been demonstrated for non-invasive imaging applications [[5], [6], [7]].

Performing both PA and OCT imaging with probes suitable for human coronary arteries presents significant miniaturization challenges. Several types of PA probes have been considered to date [[8], [9], [10], [11], [12], [13], [14]]. Typically, miniature PA catheters capable of providing volumetric images for intravascular imaging are realized by integrating optical fibers for excitation light delivery with single-element piezoelectric ultrasound (US) transducers. For circumferential imaging, rotational scanning of the excitation light can be achieved by proximal rotation of the excitation fiber [15] or by distal end rotation of a 45° reflective mirror [16]. All-optical PA probe designs with endoscopic imaging capabilities, comprising micro-rings [17] and π-shifted FBGs [18] for ultrasound detection, were previously demonstrated. An all-optical IV imaging probe that provided both PA and OCT, which included the use of an integrated MMF for PA excitation light delivery and a fiber optic heterodyne interferometer for ultrasound detection [19], was also demonstrated. Optical ultrasound detection in photoacoustics/optoacoustics was recently reviewed by Dong et al. [20] and Wissmeyer et al. [21].

Fiber-optic (FO) US sensors based on high-finesse Fabry-Pérot (F-P) cavities present several advantages in this context. As demonstrated recently [22,23], they provide high sensitivity (noise equivalent pressure of *ca.* 10 Pa in a 20 MHz measurement bandwidth) and wide bandwidths (40 MHz). Their miniature sizes are ideally suited for intravascular imaging. Here, we report the development of a fiber-optic IV imaging probe that provides both OCT and PA imaging. Our probe consists of a commercial OCT IV catheter and an integrated fiber optic US sensor with a high-finesse F-P cavity. We present an initial demonstration of its capabilities to perform 2-D circumferential imaging and multispectral photoacoustic imaging with stent and synthetic phantoms.

Photoacoustic excitation light was provided by a wavelength-tunable dye laser pumped by a frequency doubled Q-switched Nd:YVO_4_ laser (Elforlight, UK) (Fig. 1a). This laser is tunable over the range of 560 to 610 nm; its pulse repetition frequency was set to 2.8 kHz. Light was coupled into single mode fiber (SMF) using a precision fiber coupling fixture (F-915, Newport Corporation, USA) for delivery into to the OCT catheter. After coupling, the pulse energy varied from 50 nJ to 120 nJ across the wavelength range. A commercial OCT IV catheter (DragonFly OPTIS imaging catheter, St Jude Medical Ltd., UK) and console (ILLUMEN OPTIS, St Jude Medical Ltd., UK) was used for both PA and OCT imaging. This catheter (outer diameter: *ca*. 1 mm; total length: *ca*. 2 m) comprised a SMF and distal-end optics to focus and deflect the light into tissue (*ca*. 102.5° to catheter axis), which were encapsulated. This optical assembly was rotated within the fluid-filled outer tube of the catheter. The OCT catheter had a nominal axial resolution of 15 μm and a lateral resolution of *ca*. 25 μm [24]. The PA excitation light had a fluence ranging from 40 to 100 mJ/cm^2^ across the wavelength range of the pulsed laser.

**Fig. 1:**
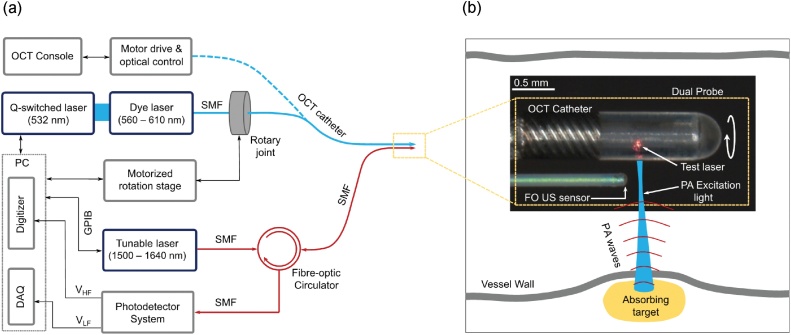
(a) Schematic of the console for the dual-modality photoacoustic (PA) and Optical Coherence Tomography (OCT) probe. PA light excitation delivery and OCT imaging are performed with an optical fiber within a commercial OCT catheter, and optical ultrasound reception is performed with a fiber optic ultrasound sensor. (b) A microscope image of the distal end of the probe, with a schematic overlay showing PA excitation light and corresponding PA ultrasound waves generated from an absorbing target within a vessel wall. The red spot is from a test laser at 632 nm. In this image, the polymer encapsulation of the OCT catheter was removed for clarity. PC: personal computer; DAQ: data acquisition; V_HF_: high frequency voltage output; V_LF_: low frequency voltage output. SMF: single-mode fiber; FO US sensor: fiber-optic ultrasound sensor.

Reception of photoacoustically-generated ultrasound was performed with a fiber optic sensor, which comprised an SMF (outer diameter: 250 μm) with a plano-concave F-P cavity at the distal end. The F-P cavity comprised a transparent polymer sandwiched between multilayer dielectric coatings, as previously described [25]. The sensor used for studies in this paper had a noise equivalent pressure of *ca.* 40 Pa, as measured with a calibrated planar transducer operating at 3.5 MHz with a 20 MHz bandwidth. The sensor had an estimated detection bandwidth of 27 MHz (−6 dB bandwidth: 3–30 MHz). Its sensitivity is nearly omni-directional across this frequency range, which allows it to receive ultrasound waves that are perpendicular to the SMF axis. Interrogation light for the fiber optic ultrasound (FO US) sensor was provided by an external cavity wavelength-tunable CW laser with a tuning range of 1500 nm–1630 nm (Tunics T100S-HP, Yenista, France). Reflected light from the F-P cavity at the distal end of the fiber was received *via* an optical circulator by a photo-receiver system with low- and high-frequency outputs. The former, which was digitized at 16 bits with a sampling rate of 250 kS/s (PCI-6323, National Instruments, UK), was used to measure the interference transfer function of the F-P cavity and to adjust the interrogation wavelength to the optimum bias wavelength of the F-P cavity. The high-frequency output, which was digitized at 8 bits with a sampling rate of 250 MS/s and a bandwidth of 125 MHz (PCI-5114, National Instruments, UK), was used to measure the PA time series (Fig. 1a). Averaging (25–50 times) across consecutive PA time series was performed for noise reduction.

The FO US sensor was positioned adjacent to the OCT catheter (Fig. 1b). Its buffer layer was affixed to the outer tube using sealing wax and epoxy. At the distal end, where the buffer layer had been removed, there was a small gap between the cladding of the FO US sensor and the outer tube of the OCT catheter (*ca*. 125 μm). The FO US sensor was aligned so that photoacoustic excitation light emerged from the distal optics of the OCT catheter slightly proximal to the F-P cavity (*ca*. 50 μm). The maximum diameter of the OCT-PA dual probe, which comprised the OCT catheter and the FO US sensor was 1.25 mm. The encapsulated SMF and distal-end optics rotated to perform circumferential imaging, whilst the FO US sensor was stationary. Rotation was performed with a motorized rotation stage (PRM1/MZ8, Thorlabs, UK) at the proximal end, with a custom built stator and rotor mounts. The data acquisition and synchronized control of the motorized rotation was performed with custom LabVIEW (National Instruments, UK) script.

The spatial resolution (PA) of the probe was estimated using optically absorbing line phantoms. For lateral resolution measurements, a carbon fiber (outer diameter: 7 μm) was imaged by translating the probe, with the probe oriented so that the translation axis and the excitation light beam were both perpendicular to the fiber. The lateral resolution was taken as the full width at half maximum (FWHM) of the maximum PA pressure time series signal received at each translation position, as estimated with Gaussian fits. With the wire positioned at depths (relative to the FO US sensor) that ranged from 0.5 mm to 2.5 mm, the lateral resolution varied from 18 μm to 40 μm (Fig. 2a). For axial resolution measurements, a tungsten wire (outer diameter: 27 μm) was imaged. The envelope of the PA time series signal was obtained and the FWHM, converted to distance (speed of sound: 1485 m/s), was taken as the axial resolution. The tungsten wire was preferable for axial resolution measurements as ringing artefacts were visually absent, but its outer diameter was too large for lateral resolution measurements. The average axial resolution across the depth range of 0.7–2.7 mm was 45 μm, with a maximum variation of ± 3.5 μm (Fig. 2a).

**Fig. 2:**
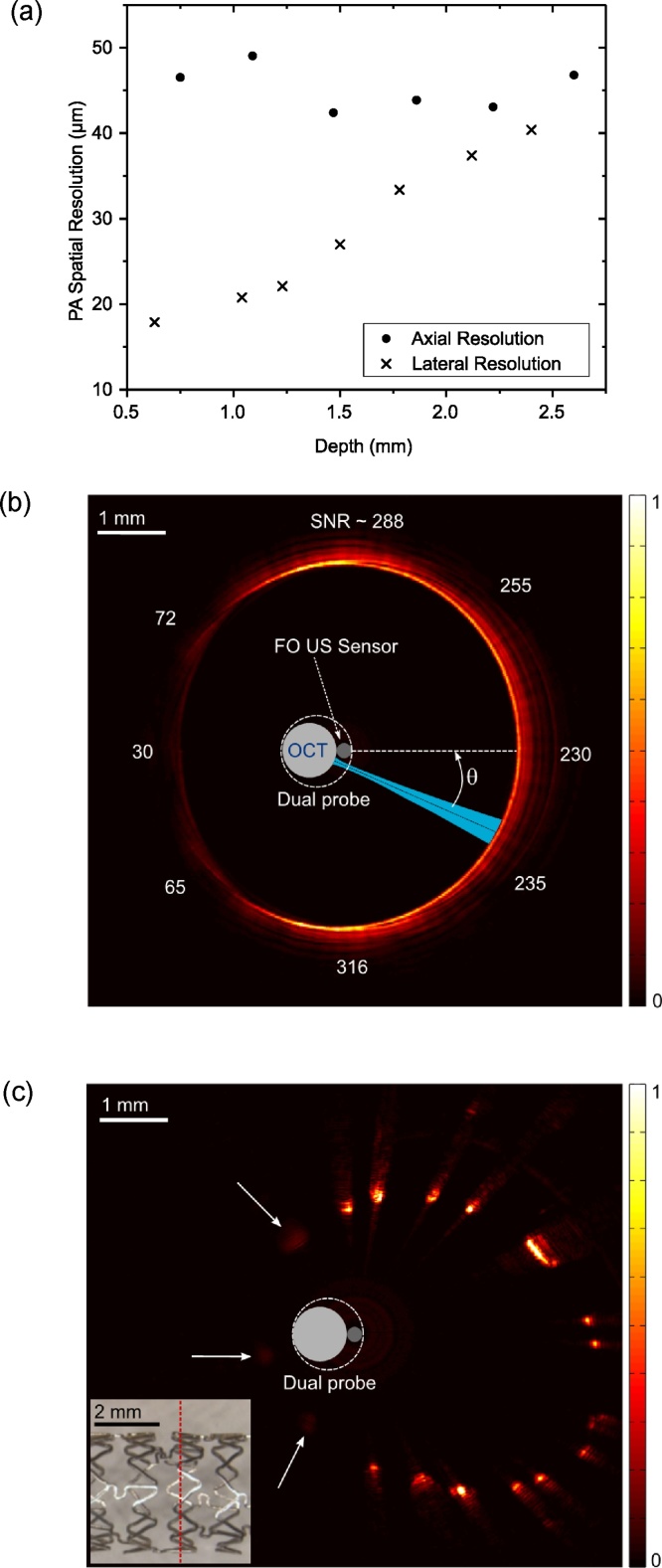
(a) Photoacoustic (PA) axial and lateral resolution of the probe estimated in the depth range from 0.5 to 2.5 mm. (b) A 2D PA circumferential image of an absorbing circular line phantom, with the signal-to-noise ratio (SNR) at different angular positions indicated. (c) A 2D PA image of a coronary stent. All of the struts are visible; those in the ultrasonic shadow of the OCT catheter (arrows) have lower signal intensities. A micrograph (inset) of the stent shows the position of the imaging plane (red dashed line). Images (b) and (c) are displayed on linear scales.

The PA signal strength varied with the angle of the photoacoustic excitation light beam relative to the FO US sensor, θ. To estimate this variation, a circular absorbing line phantom was used. This phantom was a black silicone cylinder with an inner diameter of 6 mm. The probe was positioned such that the FO sensor was at the centre of the cylinder, and imaging was performed with rotational scans (Fig. 2b). Each depth scan in an image was acquired with averaging across 50 consecutive PA time series; the magnitudes of Hilbert-transformed averaged PA time series were displayed on a linear scale in Cartesian coordinates. When the excitation light beam was directly in front of the FO US sensor (θ = 0°), photoacoustically generated ultrasound waves had a direct path to this sensor. In contrast, when the excitation light beam was in the opposite direction (θ = 180°), the ultrasound waves received by the FO US sensor had an indirect path that included propagation within the acoustically heterogeneous OCT catheter. Whilst PA signal was observed for all angles, the strength was lowest in the vicinity of θ = 180°. The signal-to-noise ratio (SNR), varied from a minimum of 30 to a maximum of 316. The highest SNR values were obtained at angles in the vicinity of θ = 90° and θ = 270°, where the fluence of excitation light at the inner surface of the cylinder may have been higher than that when θ = 0° due to the offset between the centre of the cylinder and the OCT catheter axis. An expanded coronary stent was clearly visible with PA imaging (Fig. 2c). The stent (Xience Pro, Abbott, UK) had a post-expansion diameter of 3.6 mm and a strut thickness of 125 μm. The probe was positioned inside the stent, offset from the centre. Stent struts were visible at all angles, albeit with differences in PA signal strength that were consistent with those observed with the cylinder phantom. With stent imaging, each PA time series was filtered in the Fourier domain to compensate for the frequency-dependence of the FO US sensor sensitivity. This was done by choosing the time-series signal from one of the struts with relatively high SNR (from θ = 270° region) and taking the Fast Fourier Transform of this signal as the reference to normalize the each time-series data in the Fourier domain.

A vascular phantom with inclusions was used to demonstrate multispectral PA imaging. This phantom comprised PDMS with 0.2% TiO_2_ to simulate the optical scattering of vascular tissue [26]. A wall-less cylindrical cavity with an inner diameter of 3.15 mm was created by withdrawing an acrylic tube after PDMS curing. Along the length of the cavity, two polymer micro-capillary tubes (ID/OD: 500/600 μm) to mimic inclusions were positioned. Aqueous solutions were injected into the tubes served as PA contrast agents: methylene blue (*ca*. 1.1 g/L) in one tube, and India ink (*ca*. 0.9 mL/L) in the other. The cavity and both inclusions were clearly apparent with OCT imaging (Fig. 3a). Due to strong optical absorption, a shadow was apparent beyond the inclusion with India ink. For PA imaging (Fig. 3b), the probe was positioned close to the centre of the cavity and the images were acquired with different excitation light wavelengths (λ_exc_) that ranged from 565 nm to 605 nm. Both inclusions were apparent at all PA excitation wavelengths. PA signal artefacts beyond the inclusions were apparent, which may have originated from non-uniform frequency response of the FO US sensor; the filtering performed for stent imaging did not have an appreciable effect for the vessel phantom and therefore it was not used. There were prominent differences in the wavelength-dependencies of the PA signal amplitudes obtained from the two inclusions (Fig. 3c). In particular, there was a two-fold increase in the PA signals obtained from the Methylene blue inclusion across the measured wavelength range (*cf*. a 3-fold increase in the optical absorption coefficient, as measured spectrophotometrically), whereas the PA signals obtained from the India ink inclusion were nearly constant.

**Fig. 3:**
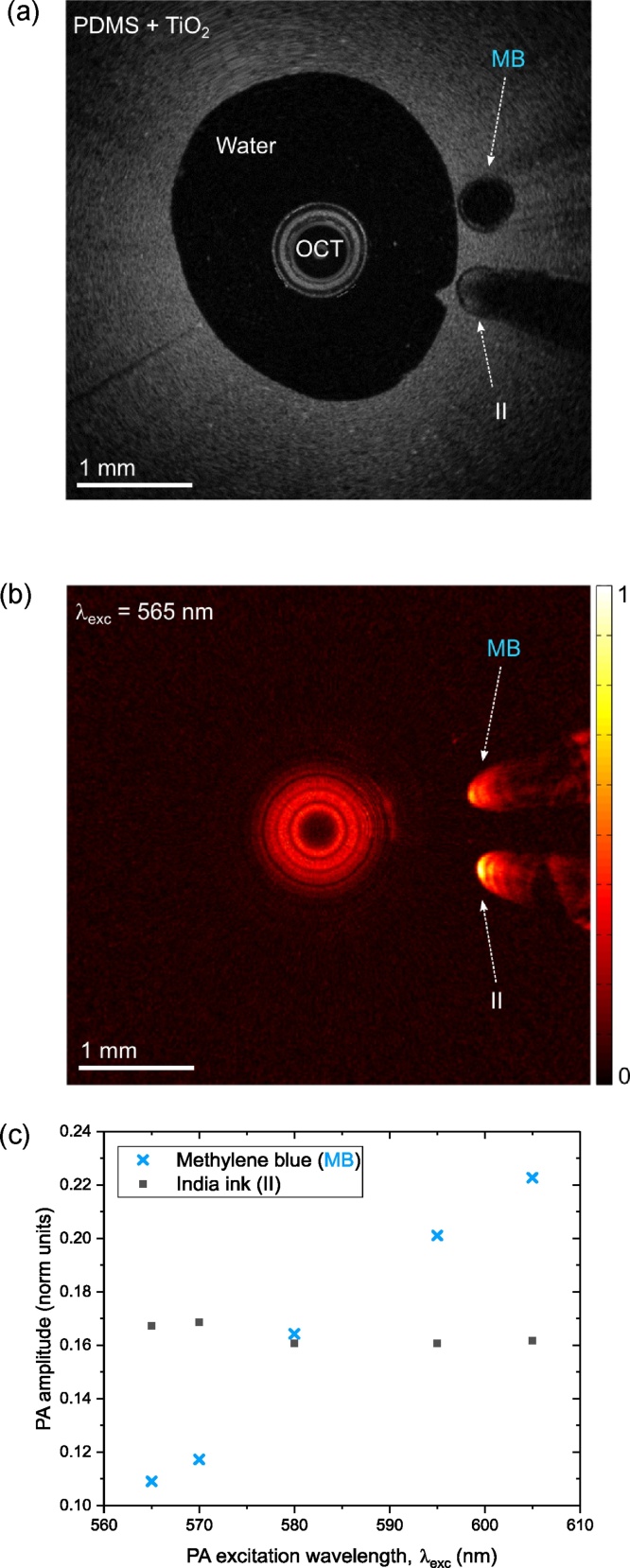
(a) OCT image of the vascular phantom with two inclusions within the wall: methylene blue (MB) within one tube and India ink (II) in another. b) A 2D photoacoustic (PA) circumferential image of the phantom at an excitation wavelength (λ_exc_) of 565 nm. c) The PA amplitude wavelength dependencies for the dyes (MB & II) estimated from PA images of the phantom acquired at multiple wavelengths in the range from 565 nm to 605 nm.

In summary, we demonstrated an all-optical, dual modality intravascular probe using a commercial OCT IV catheter and a high sensitivity fiber optic F-P cavity US sensor. To the authors’ knowledge, this is the first demonstration that a OCT catheter, including both the distal-end light-focusing optics and the proximal-end rotary components, can deliver excitation light for PA imaging. The use of a high sensitivity fiber optic US detector preserves the all-optical design of the probe; it leads to a minimal increase in lateral dimensions and does not hinder the OCT operation. Moreover, optical fibers confer immunity to electromagnetic interference, which can be important in the cardiac catheterisation room [27]. This probe was shown to provide lateral PA resolution that is similar to that of OCT imaging (18 μm–40 μm) and high PA axial resolution (*ca.* 45 μm). As with OCT, the optical focus is the governing factor for the lateral resolution of photoacoustic imaging; the frequency response of the ultrasound detector determines the axial resolution. The excitation light wavelength used for this study is outside the operational range of single-mode telecommunications fiber, which has a cut-off wavelength at *ca*. 1250 nm. As a result, there is multimode propagation of the excitation light in the wavelength range used for the current study that could increase the focused spot size of the PA excitation beam and thereby decrease the lateral resolution. The probe is likely to have a better lateral resolution with excitation wavelengths closer to the single mode operation range of the OCT fiber, for example at the lipid absorption wavelength of 1210 nm. One limitation of the current probe configuration is that a stationary fiber optic ultrasound receiver results in shielding of the PA waves by the OCT catheter for certain excitation angles. As a result, the detected PA signal amplitude varies relatively with respect to the receiving angle in the rotation plane. In future versions of the probe, an additional US sensor positioned diametrically opposite to other could eliminate the shielding effect. The rotational scanning speed of the probe in the current design is greater than 4 rotations per minute (rpm), which is limited by the speed of the motorized rotation stage. This could be significantly improved with the use of a commercial fiber optic rotatory joint, which can have a nominal rotation rate of 10,000 rpm, provided that the pulse repetition frequency of the excitation laser source was sufficiently high. Greater excitation light energies could be delivered through the inner cladding of a double clad fibre, leveraging advances in dual modality OCT/fluorescence imaging [28].

Further development of the console and probe are required to enable *in vivo* lipid imaging. As recently reviewed by Li and Chen [29], excitation light sources of 1210 and 1720 nm have been found to be useful for providing lipid contrast [[30], [31], [32], [33]], but an excitation light source at one of these wavelengths with a sufficiently rapid repetition rate and pulse energy for real-time imaging has proven elusive. For PA probes, optimizing catheter design has been the focus of several studies [[11], [12], [13], [14]], and identifying a protective sheath material that is transparent to PA and US signals has emerged as a one of the primary challenges [34]. The inclusion of OCT as an additional probe modality could impose even more stringent demands on the sheath material to minimise distortion of the OCT beam, if a single sheath were to encapsulate both the OCT probe and the FO sensor. The addition of intravascular ultrasound imaging capabilities to this probe could be valuable to provide structural contrast at depths significantly greater than those of OCT. These capabilities could potentially be added with a fiber optic ultrasound transmitter, leveraging recent advances in optical ultrasound generation with nanocomposites [35] and *in vivo* demonstrations [36]. The ability of the probe presented here to distinguish between different absorbing chromophores, by judicious choice of excitation wavelength(s), suggest that combined photoacoustic and OCT imaging could be a powerful tool to obtain functional, depth-resolved information on plaque and lipid pools inside the depth of the vessel wall to improve stent placement.

## Conflict of interest

The authors declare that there are no conflicts of interest.
